# Summer at the beach: spatio-temporal patterns of white shark occurrence along the inshore areas of False Bay, South Africa

**DOI:** 10.1186/s40462-018-0125-5

**Published:** 2018-05-22

**Authors:** Alison A. Kock, Theoni Photopoulou, Ian Durbach, Katya Mauff, Michael Meÿer, Deon Kotze, Charles L. Griffiths, M. Justin O’Riain

**Affiliations:** 1 South African National Parks, Cape Research Centre, Cape Town, 8000 South Africa; 2 South African Institute for Aquatic Biodiversity (SAIAB), Private Bag 1015, Grahamstown, 6140 South Africa; 3 Shark Spotters, P. O. Box 22581, Fish Hoek, 7974 South Africa; 4 Institute for Communities and Wildlife in Africa, Department of Biological Sciences, University of Cape Town, Private Bag X3, Rondebosch, 7701 South Africa; 5 Department of Zoology, Institute for Coastal and Marine Research, Nelson Mandela Metropolitan University, Port Elizabeth, 6031 South Africa; 6 Centre for Statistics in Ecology, Environment and Conservation, Department of Statistical Sciences, University of Cape Town, Rondebosch, 7701 South Africa; 7 African Institute for Mathematical Sciences, Cape Town, 8000 South Africa; 8 Department of Statistical Sciences, University of Cape Town, Rondebosch, 7701 South Africa; 9 Department of Environmental Affairs, Oceans and Coasts Branch, Cape Town, 8000 South Africa; 10 Department of Biological Sciences and Marine Research Institute, University of Cape Town, Rondebosch, 7701 South Africa

**Keywords:** White shark, *Carcharodon carcharias*, Telemetry, Habitat use, Marine protected area, Marine spatial planning, Conservation, False Bay, Cape town

## Abstract

**Background:**

Understanding white shark (*Carcharodon carcharias*) habitat use in coastal areas adjacent to large cities, is an important step when formulating potential solutions to the conservation conflict that exists between humans and large predatory sharks. In this study, we present the findings of a 2.5-year study of white shark occurrence and movement patterns adjacent to the City of Cape Town in False Bay, South Africa, with a focus on spring and summer months. Fifty-one white sharks were monitored annually at three offshore and twelve inshore sites by VR2 acoustic receivers, over 975 days from 1 May 2005 to 31 December 2007.

**Results:**

Occurrence patterns at inshore sites during spring and summer were analysed using a generalized additive mixed model (GAMM) with a spatial term (longitude, latitude), time of day and year included as explanatory variables for site use. We found that sharks occurred more frequently at inshore sites along the northern and northwestern shores, compared to the rest of the bay, and they transitioned most frequently between four adjacent beach sites that encompass the most popular recreational water use areas in Cape Town. There was significant diel variation, with higher shark occurrence around midday, and a peak in shark occurrence in 2005, when human-shark interactions also peaked. However, we found no effect of shark size on occurrence patterns at inshore sites.

**Conclusions:**

White sharks showed the highest levels of occurrence at specific inshore sites between Muizenberg and Strandfontein beach, and thus inclusion of these sites within False Bay’s marine protected area (MPA) network or recognition as Ecological or Biological Significant Areas (EBSAs) should be a future consideration. These insights into white shark habitat use at inshore sites in False Bay are important for successfully applying the principles of marine spatial planning (MSP) and for making science-based policy decisions. Furthermore, this information can be used to reduce potential shark-human conflict by incorporating it into future shark safety education campaigns.

**Electronic supplementary material:**

The online version of this article (10.1186/s40462-018-0125-5) contains supplementary material, which is available to authorized users.

## Background

Understanding the movement and habitat use of vulnerable marine top predators is essential for devising improved conservation and management strategies [1–3]. This is particularly important for large predatory shark species that aggregate in coastal areas threatened by diverse anthropogenic activities. Threats include intensive shore-based fishing, prey loss, pollution, culling to reduce shark bites, and the transformation or disturbance of natural habitats [4–6]. Movement and occurrence patterns of top predators can be included in marine spatial planning (MSP) e.g. to design marine protected area (MPA) boundaries or identify areas of conservation and biodiversity interest, such as Ecological or Biological Sensitive Areas (EBSAs) [7–9].

White sharks (*Carcharodon carcharias)* are apex predators that have small populations, spend considerable time near the coast and are vulnerable to human impacts due to being long-lived and having slow growth and low fecundity [10–12]. Consequently, since 2009, they have been listed as *Vulnerable* on the IUCN Red List of Threatened Species and protected in several countries [13]. Both juvenile and sub-adult white sharks use coastal areas extensively [14–21] with evidence of high fidelity at discrete beach sites in both Australia [17] and South Africa [16, 18].

In False Bay, South Africa, [20] reported both marked seasonal and sexual segregation of island versus inshore habitat use by white sharks. Both sexes aggregate around Seal Island over the austral autumn and winter, with females moving inshore in spring and summer [20]. Along the inshore regions of False Bay, potential teleost and elasmobranch prey resources are being heavily overfished [22–26] and white sharks are occasionally caught in three fisheries, namely recreational rock and surf fishing, beach purse-seine fishing and experimental fishing gear ([27], unpublished data). Thus, future MSP could benefit white sharks by conserving important prey resources, habitats and/or mitigating incidental catches [7–9].

The aims of this study are two-fold; first, to describe white shark movements between inshore and island sites year round, and second, to make inferences about the fine-scale spatial and temporal patterns of occurrence at a number of different inshore sites, during spring and summer when shark sightings peak inshore. We use passive acoustic telemetry data to test the null hypotheses that all sites along the inshore area of False Bay, South Africa are used equally, irrespective of shark size, and that there are no diel or annual differences in white shark occurrence at these inshore sites. Results from the study will improve our understanding of how a threatened apex predator uses an inshore area adjacent to a major metropole, and identify sites with higher occurrence that could be priority conservation areas. In addition, understanding where and when large predatory sharks overlap with recreational ocean users may assist relevant authorities in their goal to use non-lethal management to reduce the spatial overlap between people and sharks [28].

## Methods

### Study site

**Fig. 1:**
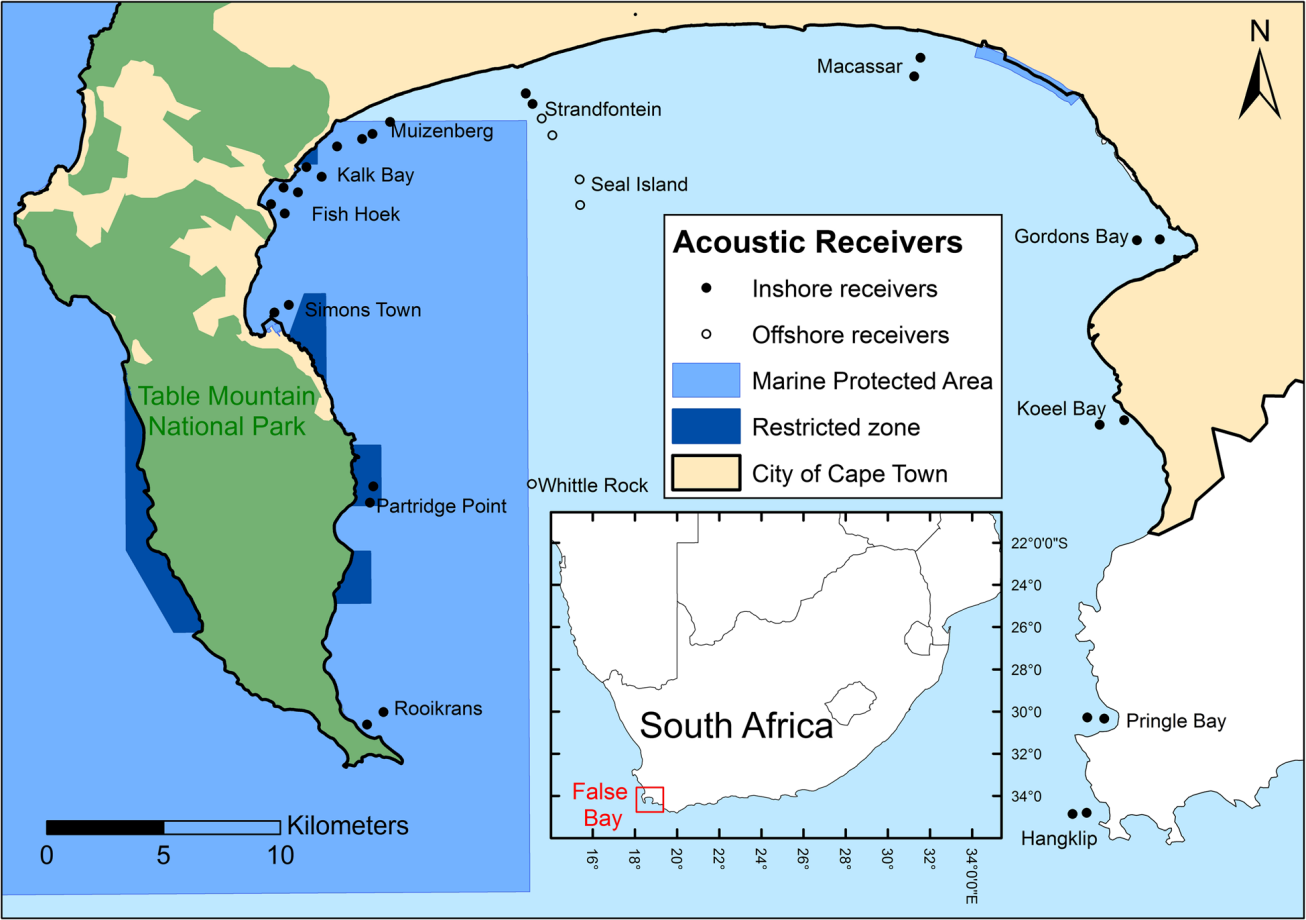
Map of False Bay, South Africa showing place names mentioned in the text and locations of inshore acoustic receivers (solid circles) used in the generalized additive mixed model (GAMM) and offshore receivers (open circles) used as reference locations in Fig. 2

This study was conducted in False Bay, on the southwestern tip of South Africa (34°04 - 34°23 S, 18°26 - 18°51E) (Fig. 1). Seal Island is located within the northern section of the bay and is home to approximately 70,000 Cape fur seals (*Arctocephalus pusillus pusillus*) [29]. Partridge Point is the only other site in False Bay where seals regularly haul-out, and while feeding is known to occur within the bay, most seals travel to foraging grounds outside of the bay [30]. Water temperature in False Bay varies seasonally from a mean summer temperature of 21.5 °C to a mean winter temperature of 13.2 °C [31]. Wind-induced upwelling occurs during spring and summer months, particularly off Cape Hangklip and Gordons Bay which results in the eastern and middle reaches of the bay having colder water compared to the northern and western regions [31, 32]. Furthermore, the shallower waters of the northern region of the bay are influenced by sun-warming during spring and summer and experience the highest temperatures in False Bay [31, 32]. The warmer water in spring and summer results in blooms of surf-zone diatoms, which are associated with an increase in abundance and diversity of teleosts and chondrichthyans [33, 34], both of which are common prey for white sharks [35, 36]. Unfortunately, site-specific water temperature and phytoplankton data were not available for the study period, as in situ data were not recorded and satellite remote sensing data were mostly missing or biased due to the proximity of the receivers to the coast [32].

The northern shore (Muizenberg to Macassar) comprises gentle-sloping, long, dissipative sandy beaches which are devoid of kelp (*Ecklonia maxima*) beds, while the eastern (Gordons Bay to Cape Hangklip) and western (Kalk Bay to Rooikrans) margins of False Bay are characterized by steep rocky shores and dense kelp beds, interspersed with small sandy bays e.g. Fish Hoek and Koeel Bay [37, 38] (Fig. 1). Two MPAs have boundaries within False Bay, namely the Table Mountain National Park MPA (TMNP-MPA) which includes restricted no-take zones and is managed by the Department of Environmental Affairs (DEA) and the South African National Parks (SANParks), and the Helderberg MPA which is a restricted zone managed by the City of Cape Town (Fig. 1).

### Tagging of sharks

White sharks were tagged at Seal Island and along the inshore region of False Bay between Muizenberg and Macassar beach (Fig. 1). The size of each tagged shark was estimated to the nearest 0.5 m using the width of the research vessel (2.6 m) as a reference. Sex was determined by visual inspection for presence or absence of claspers, and sharks were only tagged once their sex was confirmed. Acoustic transmitters were deployed into the base of the first dorsal fin using a modified spear gun. Sharks were tagged with V16-5H-R04K (69 kHz, code intervals: 150 - 300 s, 17 × 95 mm, battery life approx. 36 months) acoustic transmitters (Vemco Ltd. V16, Nova Scotia, Canada) attached to a ~ 10 cm tether and a plastic Domeier dart. This study uses the same methods of tagging white sharks in False Bay, South Africa and for more details please see [20].

### Acoustic monitoring array

An array of 33 VR2 acoustic receivers (Vemco Ltd.) was deployed in False Bay at 12 sites along the inshore region (*n* = 28 receivers) of False Bay and at three offshore sites (*n* = 5 receivers) (Fig. 1). Detection data spanned the period 1 May 2005 - 31 December 2007 with the inshore analysis restricted to spring and summer months when detection rates are highest [20]. The selection of sites allowed us to investigate white shark occurrence and visitation patterns around False Bay. Inshore sites were equipped with at least two receivers (maximum of four), with the first an average of 660 m from shore (range 230 - 1230 m) and the second an average of 1163 m meters (range 500 - 2260 m) from shore, along a straight line perpendicular to the coast (Fig. 1). Due to the wide (~ 500 m) and shallow surf zone at Muizenberg, and the steep (> 40 m) drop-off at Partridge Point, receivers were deployed horizontal (and not perpendicular) to the coast. We assigned receivers to a site, based on the distance of receivers to one another. Receivers within 2 km of each other were considered to belong to the same site. All sites had at least two receivers, with popular recreational sites having more e.g., Fish Hoek (*n* = 4), Kalk Bay (*n* = 3) and Muizenberg (n = 3). The receivers at Fish Hoek were placed very close together (< 1 km apart) so that the receiver ranges overlapped substantially, whereas all other receivers were at least 1 km apart. The effect of receivers having overlapping ranges is that sharks are often detected on more than one receiver at the same time, and the site has a smaller total range of detection. To be able to use the number of receivers at each site as a measure of its acoustic coverage in the statistical analysis (described below), we assumed that the four receivers in the Fish Hoek had the effective detection capability of only two receivers.

### Range tests

The performance of acoustic receivers in marine environments is variable and affects detection rates [39, 40]. Therefore, in situ range tests were performed on each individual receiver in the array to determine its reception range. We deployed a V16 transmitter, identical to the transmitters used in the study, over the side of the research vessel at a depth of 2 m. The boat was then moved in 50 m increments away from the receiver, using the on-board GPS system, to a maximum distance of 1200 m. The timing of the detections was matched to the distance of the transmitter from the receiver to generate a detection profile for each receiver. Range testing was conducted on relatively calm days, < 3 m swell and < 20 km/h wind.

### Statistical analysis

We first calculated the number of unique visits to each site, in each year. We considered a new visit to be two or more consecutive detections of a shark at any of the receivers at a site, provided that the shark had not been detected at the site within the previous 60 min. Single detections were excluded to reduce the impact of false detections, following [41]. White sharks are known to travel along the coast at speeds of 3 – 5 km/h [16] and the elapsed time of 60 min between visits of the same shark thus allows them to leave the detection range of the receivers at a site and potentially return.

We carried out a descriptive analysis of white shark movements between inshore and island sites throughout the year. We generated two visual representations of the transitions between sites, one showing the average number of visits to each site per 30 days of monitoring (units: n/30 days), and the other showing the total distance covered by sharks commuting directly between each pair of sites (units: km/30 days). The latter quantity is intended as a distance-standardized measure of movement activity between pairs of sites, and is equivalent to the average number of sharks detected at both sites within a given time window (with no intervening detections at other sites), weighted by the distance between sites to compensate for the higher chance of being detected at two nearby sites. To allow sharks more time to move between sites that are far apart, we set the length of the time window to the distance between the pair of receivers (in km) divided by 2 km/h (less than the known travel speeds of 3 – 5 km/h as sharks will not necessarily swim the shortest route between sites).

We fitted a generalized additive mixed model (GAMM) with the “mgcv” package in R [42] to model the dataset of counts of unique visits to all sites during spring and summer months (September to February). We fitted the model using the “gam” function with the “REML” fitting method [43], and we used the “re” smooth to include a random effect for individual shark. This accounts for the fact that individual sharks were detected repeatedly during the study period. The candidate explanatory variables included in the model were chosen on the basis of availability (longitude, latitude, size category, year, time of day), since we did not have in situ water temperature or other biological measurements available at the location of the receivers.

The final model included a spatial term (longitude and latitude in WGS84 decimal degrees, range 18.44 - 18.85 and − 34.38 - -34.089, respectively), year (integers, 2005 - 2007), time of day (integers, 0 - 23) and a random effect for individual shark (36 individuals). We explored multiple variables relating to the habitat at each site (e.g. kelp presence or absence, sandy, rocky or mixed coastline, distance to Seal Island, seafloor depth), but all of them were confounded with the spatial effect and could not be included in the model together with the spatial effect. This problem of explanatory variables being correlated with each other is common in studies such as ours, and the only practical solution is to include only one of them.

We chose to include the spatial effect in the final model over any of the habitat variables because it accounts for features that were not measured at the time of the data collection and provides a better understanding and visual representation of white shark space use along the inshore. This was also the most methodologically sensible model because the spatial effect accounts for sites being located unevenly across the bay, meaning some sites are closer together than others. We fitted the spatial effect as a two-dimensional smooth term using an adaptive basis function “ad”, with smooth dimension of 36 knots (*k* = 6) and a penalty dimension of 3 (*m* = 3). We chose an adaptive basis function to allow the fitted surface to be smoother in areas with less data and more wiggly in areas with lots of data. We fitted ‘year’ as a factor rather than a smooth variable because of only using data from a few months in each year, making the data from each year discrete. We fitted ‘time of day’ as a cyclic smooth to allow for the fact that 23h00 and 00h00 are adjacent, we used the “cc” cyclic penalized cubic regression smooth basis function with 5 knots (*k* = 5). Although the exact number of knots is not critical, this was chosen conservatively and with the intension of producing biologically meaningful results. Nonetheless, we also checked that we did not over specify the number of knots using the effective degrees of freedom as a guide [42].

The number of visits were assumed to be Poisson distributed. Counts of visits were modeled as dependent on spatial location (longitude and latitude), time of day, shark size and year. The model also included three offset terms, to account for variation in the number of receivers at each site (range 2 - 4), the maximum receiver range for each site (range: 0 - 1, where 0 ≤ 500 m and 1 > 500 m), and the number of months for which there were observations in each year (2005: 4, 2006: 6, 2007: 6).

In “mgcv” it is possible to do automatic variable selection by letting the model fitting procedure shrink the effect of a covariate to zero, effectively removing it from the model, by setting the “select” argument equal to true (“double penalty” approach) [44, 45]. This is the approach we used to do model selection, starting with a sensible, minimal model that was led by specific hypotheses. Some of the advantages of this approach over the ‘all subsets’ approach are described by [46]. We checked that the assumptions of the model were met by examining residual and random effects diagnostic plots. All statistics were carried out using R software (version 3.3.2; R Core Team).

## Results

### Sex and size of tagged sharks

A total of 53 white sharks were tagged with acoustic transmitters in False Bay between 1 May 2005 and 31 December 2007 (2005, *n* = 23; 2006, *n* = 25; 2007, *n* = 5). Additionally, three sharks tagged in 2004 at Seal Island as part of a long-term study, returned in 2005 and were included in the analysis, bringing the total number of acoustically monitored sharks for the study period to 56. Of these, 51 sharks were included in the analysis of movement between sites (five sharks were excluded from the analysis because they left the study area soon after tagging), and 36 sharks were detected on the False Bay receiver array during spring and summer months between 1 May 2005 and 31 December 2007 and included in the GAMM analysis (Table 1). Most sharks (80%) were tagged at Seal Island with only 20% tagged in the inshore region between Strandfontein and Muizenberg (Table 1). Inshore tagging was only conducted during the summer of 2006/2007 and only female sharks were encountered in the 11 tagging trips. The median size of all tagged sharks included in the analysis (*n* = 51) was 340 cm TL, while the median size of tagged sharks detected at inshore sites only (*n* = 36) was 330 cm TL. Sharks fell predominantly into the > 3 m category (71%) and were mostly female (69%). Tagged animals in this study (based on an estimated size at maturity of > 350 cm TL for males and > 450 cm TL for females [39]) represent mostly juveniles and sub-adults.

**Table 1:** Summary of tag deployments on white sharks *Carcharodon carcharias* detected in False Bay between 1 May 2005 and 31 December 2007. White sharks which are likely mature (based on their size) are highlighted in bold

Shark-ID	TL (cm)	Size category	Sex	Area tagged	Date tagged	Date of last acoustic detection in False Bay	Monitoring period (days)	No. of days detected in False Bay
Included in statistical analysis (GAMM)								
545	280	≤3	F	Inshore	11/14/06	12/31/07	413	180
546	280	≤3	F	Island	04/28/06	12/27/06	244	210
547	350	> 3	F	Island	06/30/06	07/27/07	393	282
548	320	> 3	F	Island	04/28/06	11/04/07	556	164
549	300	≤3	F	Island	08/17/06	07/21/07	339	185
551	320	> 3	F	Inshore	11/14/06	11/06/07	358	149
553	340	> 3	M	Island	06/30/06	08/01/07	398	110
556	380	> 3	F	Island	08/09/06	10/30/06	83	76
557	280	≤3	M	Island	08/17/06	10/24/06	69	40
558	370	> 3	F	Inshore	10/06/06	02/27/07	145	110
560	170	≤3	F	Inshore	11/13/06	04/17/07	156	129
562	340	> 3	F	Inshore	11/14/06	05/23/07	191	181
**601**	**450**	**> 3**	**F**	**Island**	**08/25/05**	**09/16/05**	**23**	**21**
603	380	> 3	F	Island	05/20/05	01/03/06	229	169
**604**	**350**	**> 3**	**M**	**Island**	**08/29/05**	**09/21/06**	**389**	**164**
605	320	> 3	M	Island	08/24/05	11/07/06	441	67
608	360	> 3	F	Island	06/04/05	10/03/05	122	84
611	250	≤3	F	Island	09/02/05	05/07/06	248	151
612	220	≤3	M	Island	05/19/06	06/15/07	393	74
615	320	> 3	M	Island	08/30/05	06/18/06	293	22
620	360	> 3	F	Island	06/17/05	12/27/05	194	180
621	300	≤3	F	Island	06/06/05	11/17/05	165	126
622	340	> 3	M	Island	06/10/05	10/18/05	131	60
623	330	> 3	F	Island	06/10/05	01/22/06	227	60
626	250	≤3	F	Island	06/10/05	01/08/06	213	149
630	340	> 3	F	Island	05/25/06	09/29/06	128	65
632	300	≤3	F	Inshore	11/13/06	01/28/07	77	61
633	330	> 3	F	Inshore	01/26/07	11/08/07	287	257
634	380	> 3	F	Inshore	11/14/06	04/17/07	155	135
635	300	≤3	F	Inshore	11/14/06	12/29/06	46	37
636	300	≤3	F	Inshore	11/14/06	11/24/06	11	11
637	400	> 3	F	Inshore	01/17/07	08/08/07	204	147
638	400	> 3	F	Island	03/10/07	11/02/07	238	169
639	300	≤3	F	Island	06/12/07	12/31/07	203	179
642	340	> 3	F	Island	09/14/07	11/03/07	51	47
607	350	> 3	F	Island	06/17/05	01/14/06	212	153
Additional sharks included in descriptive analysis of seasonal movements								
28	300	≤3	F	Island	09/03/04	08/31/05	363	22
**520**	**400**	**> 3**	**M**	**Island**	**04/25/04**	**08/09/05**	**472**	**69**
521	370	> 3	F	Island	04/25/04	06/13/05	415	103
533	340	> 3	F	Island	04/06/06	06/15/06	71	44
534	330	> 3	M	Island	04/06/06	08/06/06	123	88
552	250	≤3	M	Island	06/30/06	07/13/06	14	552
554	340	> 3	M	Island	07/03/06	08/18/06	47	554
606	350	> 3	F	Island	06/04/05	06/10/05	7	606
**609**	**360**	**> 3**	**M**	**Island**	**06/04/05**	**08/19/05**	**77**	**64**
610	420	> 3	F	Island	06/04/06	06/23/06	20	7
613	320	> 3	M	Island	06/28/05	09/16/06	446	71
614	360	> 3	F	Island	06/06/05	07/23/05	48	28
**624**	**500**	**> 3**	**F**	**Island**	**06/06/05**	**06/10/05**	**5**	**5**
625	300	≤3	F	Island	06/10/05	08/01/05	53	16
628	330	> 3	M	Island	05/21/06	06/08/06	19	17

### Shark occurrence and seasonal movement

**Fig. 2:**
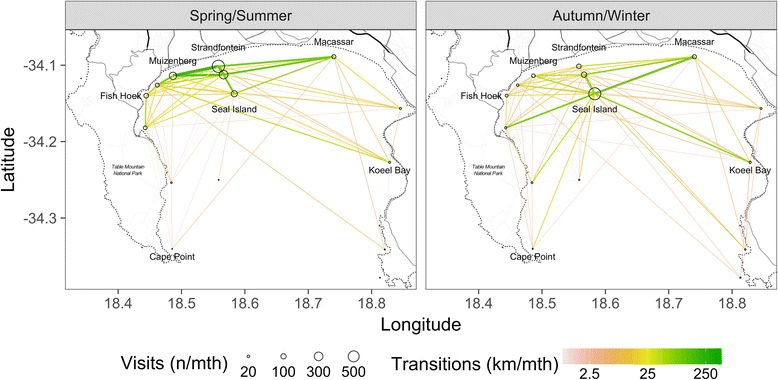
Seasonal differences in the frequency with which white sharks are observed at sites in False Bay, South Africa (total visits), and the frequency of transitions between sites (transitions). Visits to sites are denoted by open circles with the area proportional to the number of visits received (units: total visits/30 days). Transition frequencies are denoted by the colour and thickness of the line between sites (units: km/30 days). Results are shown for spring/summer and autumn/winter

A total of 89,577 detections were recorded over spring and summer months at the 12 inshore sites (Table 2, mean 7464, median 1956, range 14 to 33,913), with a median of 83.5 (range 2 - 288) days of detection per site. Tagged sharks were detected at all sites along the inshore region of False Bay with a median of 16 (range 2 - 30) individuals detected per site. Shark occurrence varied considerably by site with the highest occurrence on the northern shore of False Bay (Muizenberg and Strandfontein sites, Table 2 and Fig. 2), and the lowest occurrence along the eastern (Hangklip and Pringle Bay) and western (Rooikrans and Partridge Point) headlands. The site with the highest shark occurrence was Strandfontein, with a maximum of 12 tagged sharks detected on a given day and with sharks detected for 288 days (80%) of a 362-day monitoring period. This site also recorded the highest number of consecutive days that sharks were detected (95 days), the highest number of consecutive days that the same shark was detected (22 days), and the longest average number of consecutive days that sharks were recorded as present at the site (3.18 days). Detection statistics were highly correlated, in the sense that areas with more detections also recorded more unique sharks and longer residence periods (Table 2). Several of the sites (notably Hangklip, Rooikrans, Pringle Bay) recorded only a few brief visits. Detection statistics for autumn/winter months are provided for completeness in the (Additional file 1).

**Table 2:** Summary of raw detection data in spring and summer months at each site over the study period 1 May 2005 to 31 December 2007, sorted by frequency of detection (^a^ = Offshore sites)

Site	Monitoring days	Days with a detection	Detections	Unique sharks detected	Max unique sharks in a day	Max consecutive detection days (any shark)	Max consecutive detection days (same shark)	Mean consecutive detection days (same shark)
SFB	362	288	33,913	26	12	95	22	3.18
MSZ	484	294	21,054	28	6	63	15	1.76
KLB	484	243	12,467	30	5	30	15	1.63
FH	484	158	9981	24	4	12	5	1.57
ST	484	129	7524	22	4	17	8	1.52
MI	484	154	3316	28	4	8	7	1.40
GB	484	38	596	10	2	6	3	1.39
KB	484	24	348	10	2	2	2	1.17
PP	484	18	297	8	2	3	3	1.27
PB	303	5	37	5	1	1	1	1.00
RK	484	4	30	4	1	1	1	1.00
HK	303	2	14	2	1	1	1	1.00
SFA^a^	484	391	25,890	33	9	106	19	2.44
SI^a^	484	262	10,778	35	6	21	11	1.91
WR^a^	484	4	14	1	1	2	2	1.33

*Labels*: *RK* Rooikrans, *PP* Partridge Point, *ST* Simonstown, *FH* Fish Hoek, *KLB* Kalk Bay, *MSZ* Muizenberg, *SFB* Strandfontein, *MI* Macassar, *GB* Gordons Bay, *KB* Koeel Bay, *PB* Pringle Bay and *HK* Cape Hangklip

The strong seasonal shifts in use of the offshore and inshore habitats are summarized descriptively in Fig. 2. An additional video file with an accompanying explanation shows this in more detail (Additional files 2 and 3). In autumn and winter, activity is focused at Seal Island (602 detections/month) with primary movement activity between the offshore Strandfontein and Seal Island receivers (128.5 km/month from 40.4 transitions) and between inshore receivers and either Seal Island or, less frequently, the offshore Strandfontein receiver (Macassar/Seal Island (137.1 km/month from 8.9 detections; inshore/offshore Strandfontein 24.3 km/month from 17.0 transitions; Simonstown/Seal Island 81.4 km from 5.9 transitions; Muizenberg/offshore Strandfontein 41.6 km/month from 5.6 transitions). Movement involving inshore receivers is relatively uncommon (Fish Hoek/Kalk Bay: 13.9 km/month from 6.2 detections; Muizenberg/Kalk Bay: 12.7 km/month from 4.8 transitions).

Spring and summer activity is focused on inshore and offshore Strandfontein receivers (638 and 296 detections/month respectively), with substantial activity along all receivers on the northern shore of False Bay (Muizenberg, 206 detections/month; Fish Hoek, 75 detections/month; Macassar 55 detections/month; Kalk Bay 54 detections/month; Simonstown 45 detections/month) as well as at Seal Island (140 detections/month). The primary movement patterns are between inshore and offshore Strandfontein receivers (200.1 km/month from 140.0 transitions), between receivers along the northern shore of False Bay between Fish Hoek and Macassar, with activity decreasing with distance from Strandfontein. Sharks continue to move between both Strandfontein receivers and Seal Island (Seal Island/Strandfontein (offshore): 96.6 km/month from 30.4 transitions; Seal Island/Strandfontein (inshore): 72.6 km/month from 15.7 transitions), and between inshore receivers and both inshore and (less commonly) offshore Strandfontein receivers (e.g. Muizenberg/Strandfontein (offshore): 156 km/month from 21.2 transitions; Muizenberg/Strandfontein (inshore): 239 km/month from 35.4 transitions).

### Inshore occurrence patterns

**Fig. 3:**
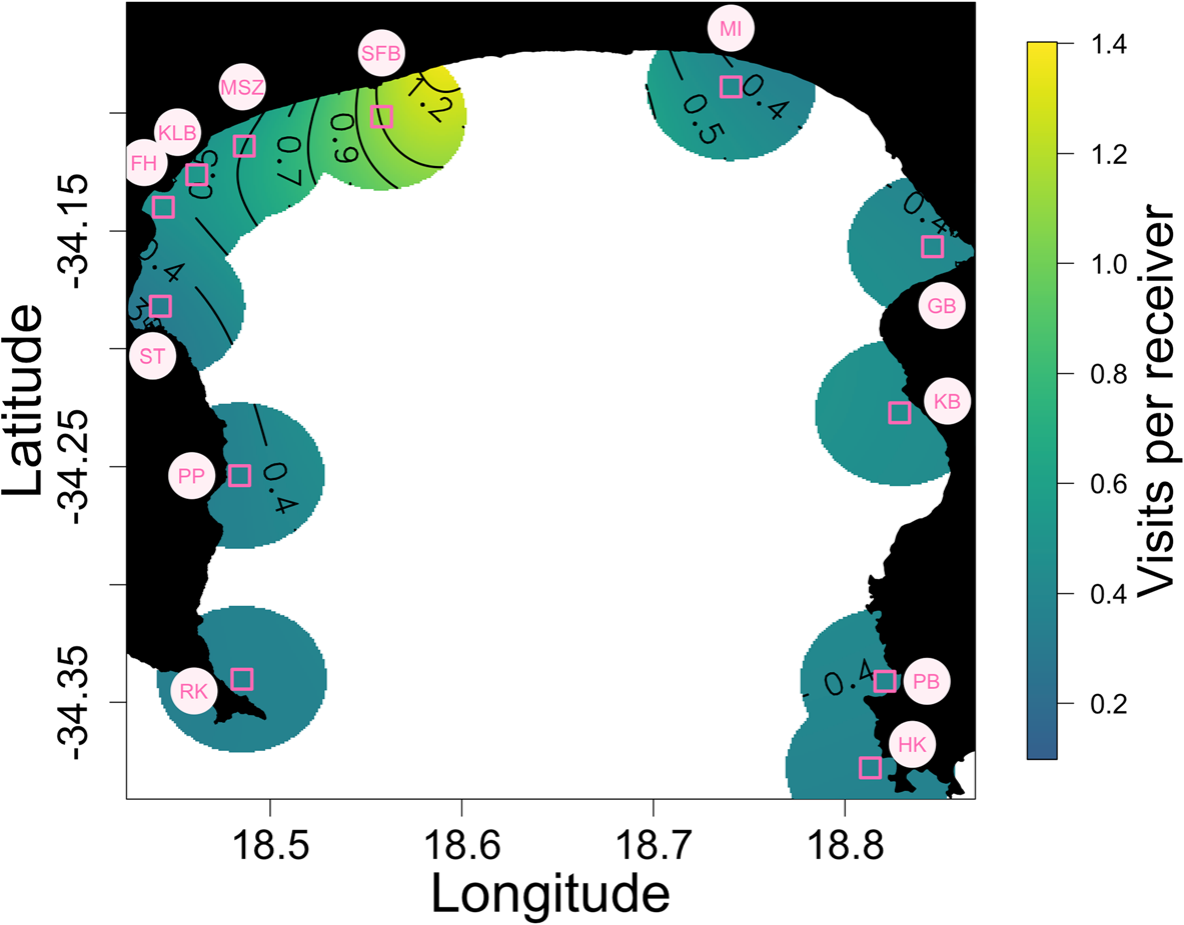
Map of model predictions for the number of visits to a single receiver at midday (13h00) in 2005, across False Bay, South Africa. These values of time and year were chosen for illustration. Predictions extend 8% of the range of the data in both directions (longitude and latitude) to avoid predicting too far from the observations. The sites are marked on the map as squares and labeled as follows: RK Rooikrans, PP Partridge Point, ST Simonstown, FH Fish Hoek, KLB Kalk Bay, MSZ Muizenberg, SFB Strandfontein, MI Macassar, GB Gordons Bay, KB Koeel Bay, PB Pringle Bay and HK Cape Hangklip

**Fig. 4:**
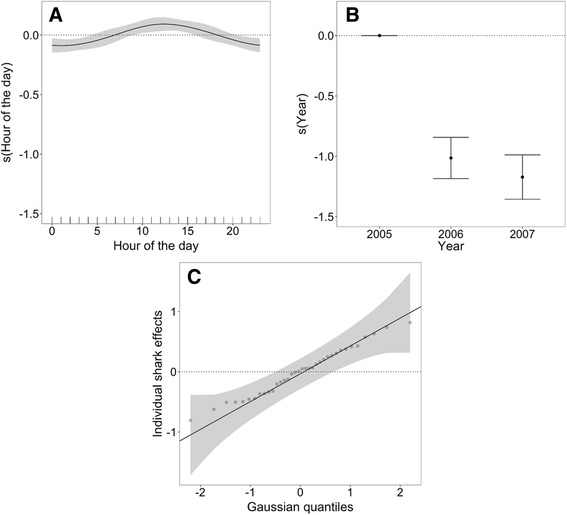
Fitted smooth relationships between the explanatory variables in the model and the number of visits to each inshore site in False Bay, South Africa in each year of the study between 1 May 2005 and 31 December 2007. These include (**a**) the smooth term for time of day, (**b**) the factor variable for calendar year and (**c**) the random effect for individual shark

Although some important explanatory variables were likely missing from the model, it nonetheless showed some clear trends in terms of the temporal and spatial distribution of white shark occurrence in False Bay during spring and summer. The component smooth functions of the explanatory variables show the relationships with shark occurrence according to the fitted model (Fig. 3, Fig. 4a-c). The approximate *p-values* for all explanatory variables (year, shark ID, time of day and spatial location) were smaller than 0.001.

These results indicate that we cannot accept the null hypotheses that all inshore sites, time of day and years are used equally by white sharks. Sites on the north and northwestern shore of False Bay (Strandfontein and Muizenberg) had a substantially higher number of visits than other sites along the northern shore or eastern and western shores (Fig. 3). This pattern was consistent across all years. There was a diurnal effect, with occurrence being higher during the middle part of the day (Fig. 4a). Despite having the fewest number of months of data, there was a clear peak in 2005, followed by a decline in 2006 and 2007 (Fig. 4b). Lastly, there were no large outliers in the distribution of individual effects (Fig. 4c). The final model explained approximately 53% of the variability in the data (model deviance). The residuals were roughly symmetrical, with a slight right skew, suggesting that the model under predicts a bit. For most individuals there was no autocorrelation in the model residuals, but there was slight positive autocorrelation in the data for a couple of individuals. It is not recommended to predict outside the range of the data used to build a model.

## Discussion

In a previous related study, [20] demonstrated a clear seasonal shift in white shark occurrence from Seal Island in autumn and winter, to the inshore region of False Bay in spring and summer. In this study, we expanded on these findings by describing the fine-scale spatial and temporal patterns of occurrence at inshore sites. Our results demonstrate clear spatial and temporal patterns of white shark occurrence, with the highest occurrence along the northern and northwestern shores of False Bay, most movement between four adjacent beach sites, most visits at midday, and in 2005. The significance of these findings and their potential application in MSP and shark safety campaigns are discussed below.

White sharks visited the northern and northwestern shore of False Bay significantly more often than sites on the eastern or western shores during spring and summer (Figs. 2 and 3). The high white shark occurrence at Strandfontein is noteworthy, with up to 12 tagged sharks (33% of tagged sharks detected inshore) recorded on a single day and any tagged shark detected at the site for up to 95 consecutive days, and up to 22 consecutive days for the same shark. There are very few seals seen at Strandfontein and Muizenberg beaches in spring and summer [30] and thus preference for this site and adjacent inshore sites, must be driven by factors other than seal presence, unlike for Seal Island. The marked preference of white sharks for distinct inshore sites along the northern and northwestern beaches of False Bay, supports the findings of [16, 18] who actively tracked white sharks in Mossel Bay, South Africa and reported prolonged visits to similar distinct beach habitats. The reasons for visits to these habitats, which are dominated by open beach habitat, remain unclear, with [16] suggesting it is used primarily for resting and socializing (based on the slow rate of movement recorded), in between more active foraging visits to a nearby seal colony. By contrast, both [17, 47] suggest that juvenile white sharks are using the inshore beach habitat primarily for foraging, with the former proposing an energy-conserving ‘sit and wait’ foraging strategy to encounter passing shoals of seasonally abundant fish that migrate along the inshore. White sharks can switch between a ‘sit-and-wait’ and ‘active searching’ (patrolling) movements when hunting [48], which could explain the high movement rates and large number of consecutive days present at sites between Fish Hoek and Strandfontein. Furthermore, studies have demonstrated a negative relationship between the level of habitat complexity and predator foraging success in aquatic environments [49, 50]. Rocky areas with dense kelp beds, like those along the eastern and western regions of False Bay, provide refugia for prey and impede the movement of large, fast swimming sharks which prefer to forage in uncluttered habitats [50], such as the open water around Seal Island [51], or in this case the sandy substrates typical of coastal beaches such as Strandfontein. Similar findings are available for terrestrial ambush predators, like lions, which track the seasonal distribution and abundance of their preferred prey, but on a fine-scale select habitat where prey is easier to catch, rather than areas where prey densities are highest [52].

White sharks may also be attracted to the warmer waters of the northern sites of False Bay [31, 32] with two studies showing a positive association between white shark sightings and warmer waters (≥18 °C) along the inshore [53, 54]. These studies propose that the association is likely due to an increase in prey availability, rather than thermal preference for warmer water at such a narrow temperature range (18 – 22 °C), as white sharks are often detected across a wider temperature range (13 – 25 °C) and can tolerate even colder temperatures [55–57]. In support of this is that white sharks are common at Seal Island, False Bay in winter when the average water temperature is 8 °C colder than the average summer water temperature [31]. However, fish and elasmobranch prey species have been confirmed to be more abundant along the inshore areas of False Bay during spring and summer, especially in the northern regions of False Bay when the water is warmer [32–34, 58]. Strandfontein in particular is a very well-known fishing location for various line-fish species e.g. kob (*Argyrosomus spp.*) and smooth hound sharks (*Mustelus mustelus*). Professional shark spotters [28] also record large schools of fish moving along the inshore in spring and summer months and the probability of detecting a white shark at both Fish Hoek and Muizenberg beaches has been shown to be significantly higher when prey fish are present [54]. Additionally, the warmer waters along the northern and northwestern shores may also facilitate a net positive energy budget for sharks, especially when prey is patchy, because they need to expend less energy in warmer waters [55, 59].

The findings that tagged white sharks spent considerable time at sites that are dominated by sandy beach habitat, high movement between Fish Hoek and Strandfontein, their generally high energy requirements [59], direct observations of foraging behavior close to beach habitat and the co-incidence of migratory prey fish along the inshore, suggest that white sharks are actively feeding in these areas dominated by open, beach habitat. However, it is clear that new methods, including cameras attached to animals (e.g. Crittercam [60]), stable isotope analysis and surveys of prey availability at different sites are needed to support or refute the hypothesis that beach habitat is preferred in False Bay during spring and summer because it facilitates foraging on migratory fish species. Future research should aim to collect in situ water temperature and associated information on prey availability at these sites.

We hypothesized that small white sharks (< 3 m), might use inshore habitats differently to larger (> 3 m) white sharks because they do not predate on Cape fur seals as frequently [35, 36]. Partridge Point (located on the western region of False Bay) is the only inshore site in False Bay where seals regularly haul-out [30] and when seals are observed along the inshore it is usually in small groups next to kelp forests along the western or eastern areas of False Bay and rare to see them along the northern shore (A. Kock, *personal observation*). However, we found that the number of visits to Partridge Point and movement to this site was very low for both size categories, suggesting that it is used to transit through, rather than hunt seals. Overall, we found that white sharks in both size categories preferred visiting and moving between sites on the northwestern and northern shore, with no size segregation observed in our study.

Understanding white shark spatial and temporal patterns at inshore sites in False Bay is important when applying the principles of MSP and decision-making regarding policy. The inshore region of False Bay is heavily impacted by fishing [22–25]. While the Cape fur seal population in False Bay seems to be stable [29], the same cannot be said for coastal fish populations [24, 26] and other shark populations in False Bay e.g. soupfin sharks (*Galeorhinus galeus*) [25]. It is possible that loss or changes in distribution of prey could impact the distribution, and spatial and temporal movements of white sharks in False Bay, in addition to inadvertently driving sharks to seek alternative prey sources. It has been demonstrated that marine reserves can benefit marine megafauna, and that megafauna can help establish target areas and boundaries for ecosystem reserves [7]. This study has confirmed that white sharks have very high levels of occurrence at Muizenberg and Strandfontein in particular and thus inclusion of these sites within False Bay’s MPA network or identification as EBSAs should be a future consideration. Such a move would increase monitoring and control of activities which may threaten white sharks or their prey resources (e.g. beach purse-seine fisheries, line-fisheries and shore angling) in False Bay. Sandy beach habitats and associated ecosystems are under-represented in the current MPA network, thus inclusion of these sites would increase the amount of sandy habitat conserved. It is also important to address potential threats to white sharks along the inshore. Perhaps highest on the list of known threats is the capture of white sharks by shore-based fisherman, either deliberately or as by-catch when fishing for other shark species. The banning of capture gear e.g. drones, large hooks, large baits and steel traces in areas with high white shark occurrence (e.g. Strandfontein) may reduce these risks, although the enforcement of such bans remains problematic. Although the data for this study was collected 10 years ago, current monitoring of the white shark population using telemetry and observational data indicates that the patterns identified here are consistent with present day observations ([28, 61] Shark Spotters, unpublished data). Resistance to extending the MPA to include these sites may come from both commercial and recreational users of these sections of False Bay and hence this proposal will have to be subject to a detailed risk assessment and extensive consultation with all of the relevant stakeholders.

The spring and summer spatial (inshore) and temporal (diel and annual) peaks in white shark occurrence have implications for both humans and sharks. We found a marked variation in the mean total number of annual visits, which peaked in 2005. This peak in shark sightings coincided with a spate of shark bites in False Bay with incidents reported in Fish Hoek and Muizenberg [53, 62]. White shark occurrence is highest in spring and summer which corresponds with the annual peak in human recreational activities and movement is highest between Fish Hoek and Strandfontein, which includes False Bay’s most popular swimming and surfing beaches [28]. Since 2000, 13 white shark bites, of which four (33%) were fatal, have been recorded on water users in False Bay, with Fish Hoek beach having the most bites (50%) and fatalities (75%) (Shark Spotters, unpublished data). Although rare, shark bites put tremendous pressure on management authorities like the Department of Environmental Affairs, Oceans and Coasts Branch and the City of Cape Town municipality, to implement lethal control programs e.g. drums lines or gill nets like those employed along the KwaZulu-Natal coast [63, 64]. Cape Town municipality has, however, opted to support a non-lethal policy at this stage [63] which aims to reduce the spatial overlap between sharks and recreational beach users through both a Shark Spotter programme [28], and an exclusion net at Fish Hoek beach (22 March 2013 Media Release, online at: www.capetown.gov.za) and through improved education and awareness of high risk sites and times. Our results indicate that current shark safety services are located at some of the sites with the highest shark occurrence (Fish Hoek to Muizenberg), but that the area between Muizenberg to Strandfontein is a high risk area which needs further attention. The information from our study should be incorporated into shark safety education campaigns, especially where shark spotters or exclusion nets are not available, so that individuals using these areas for recreational ocean activities better understand their personal risk when compared to using other areas around False Bay.

## Conclusions

White sharks are capable of extreme migrations across ocean basins [10], yet exhibit fine-scale preferences for specific sites, for prolonged periods of time. In False Bay, white sharks moved between, and visited four specific inshore sites along the sandy beach, north and northwestern areas of the bay, significantly more than other inshore sites. This information can be used to interrogate the effectiveness of existing MPA boundaries, help design new MPA boundaries or identify EBSAs. White sharks do not benefit much from the current MPA network in False Bay, as high occurrence sites, such as Strandfontein and Seal Island are not included in the network. Our results suggest that they could benefit in two different ways if these sites were included in the future. Firstly, to conserve important prey resources and secondly, to reduce being caught incidentally by fisheries. An enhanced understanding of white shark spatial and temporal patterns can also be used in shark safety and awareness campaigns to reduce the spatial and temporal overlap of people and sharks in this region.

## Additional files

Additional file 1: **Table S1.** Summary of raw detection data in autumn and winter months at each site over the study period 1 May 2005 to 31 December 2007. (DOCX 17 kb)

Additional file 2: Visualising shark detections: explanation of additional video. Provides a short explanation for viewing the video file. (DOCX 375 kb)

Additional file 3: Visualising shark detections: video. Provides a visualisation of the detection data for the study period. (MP4 13,117 kb)

## Abbreviations

DEA: Department of Environmental Affairs
EBSA: Ecological or biological significant area
GAMM: Generalized additive mixed model
MPA: Marine protected area
MSP: Marine spatial planning
SANParks: South African National Parks
TMNP-MPA: Table Mountain National Park MPA
