# Supplementary material for: Summer at the beach: spatio-temporal patterns of white shark occurrence along the inshore areas of False Bay, South Africa
Source: Mov Ecol. 2018 May 22;6:7. doi: 10.1186/s40462-018-0125-5 (PMC5963061; doi:10.1186/s40462-018-0125-5)
Supplement: Supplementary file 1 — Table S1. Summary of raw detection data in autumn and winter months at each site over the study period 1 May 2005 to 31 December 2007. (DOCX 17 kb) [file 40462_2018_125_MOESM1_ESM.docx]

**Additional file 1**

**Table S1.** Summary of raw detection data in autumn and winter months at each site over the study period 1 May 2005 – 31 December 2007 (* = Offshore sites). Rows are shown in the same order as Table 3. Labels: RK Rooikrans, PP Partridge Point, ST Simonstown, FH Fish Hoek, KLB Kalk Bay, MSZ Muizenberg, SFB Strandfontein, MI Macassar, GB Gordons Bay, KB Koeel Bay, PB Pringle Bay and HK Cape Hangklip.

| Site | Monitoring days | Days with a detection | Detections | Unique sharks detected | Max unique sharks in a day | Max consecutive detection days (any shark) | Max consecutive detection days (same shark) | Mean consecutive detection days (same shark) |
| --- | --- | --- | --- | --- | --- | --- | --- | --- |
| SFB | 342 | 82 | 1602 | 24 | 6 | 7 | 7 | 1.47 |
| MSZ | 491 | 108 | 3012 | 21 | 4 | 10 | 4 | 1.30 |
| KLB | 491 | 86 | 2119 | 25 | 6 | 7 | 7 | 1.27 |
| FH | 491 | 70 | 2206 | 23 | 4 | 6 | 4 | 1.23 |
| ST | 491 | 107 | 4077 | 24 | 5 | 8 | 8 | 1.46 |
| MI | 491 | 144 | 3039 | 31 | 6 | 14 | 6 | 1.44 |
| GB | 491 | 31 | 829 | 10 | 3 | 5 | 4 | 1.40 |
| KB | 491 | 44 | 605 | 21 | 2 | 6 | 4 | 1.24 |
| PP | 491 | 39 | 386 | 17 | 2 | 4 | 2 | 1.03 |
| PB | 311 | 11 | 244 | 10 | 2 | 1 | 1 | 1.00 |
| RK | 491 | 9 | 71 | 7 | 2 | 2 | 1 | 1.00 |
| HK | 311 | 9 | 43 | 8 | 2 | 1 | 1 | 1.00 |
| SFA* | 491 | 265 | 8205 | 39 | 7 | 17 | 10 | 1.75 |
| SI* | 491 | 438 | 97006 | 50 | 13 | 137 | 40 | 3.86 |
| WR* | 426 | 10 | 97 | 6 | 1 | 3 | 2 | 1.11 |
