# Supplementary material for: Summer at the beach: spatio-temporal patterns of white shark occurrence along the inshore areas of False Bay, South Africa
Source: Mov Ecol. 2018 May 22;6:7. doi: 10.1186/s40462-018-0125-5 (PMC5963061; doi:10.1186/s40462-018-0125-5)
Supplement: Supplementary file 2 — Visualising shark detections: explanation of additional video. Provides a short explanation for viewing the video file. (DOCX 375 kb) [file 40462_2018_125_MOESM2_ESM.docx]

**Additional file 2**

*Visualizing shark detections: explanation of additional video*

The accompanying video provides a visualization of shark movement in False Bay. Each frame of the video is a snapshot of detections and transitions recorded in the previous six hours (construction of detections and transition are described in the section *Shark presence and seasonal movement*). Each shark is displayed in a unique colour, although with 54 sharks some colours are difficult to distinguish visually. Detections at a site are shown as a filled circle at the site location. The size of the circle increases with the time that the shark has been detected at a site, up to a maximum of four hours (otherwise the size of points can obstruct information at other sites). Transitions between sites are shown as line segments between a pair of sights. Both site detections and transitions fade over time. Detections at a site, and transitions from that site, fade to transparent over a six-hour period from the last detection. The current time is shown on the video.

Figure 1 shows two consecutive frames from the video as an illustration. The first frame shows one shark moving between Strandfontein and Muizenberg, another at Simonstown, and a several other sharks with recent activity at Seal Island. In the second image, the increased size of the circle at Simonstown shows that the shark previously detected there has remained at the site. The additional transition from Muizenberg to Kalk Bay provides information on the direction of movement and is indicative of a common “inshore” movement described in this paper. The lack of change at Seal Island indicates that sharks are no longer at the site (the filled circles have faded marginally, but this will be difficult to see in the accompanying image).


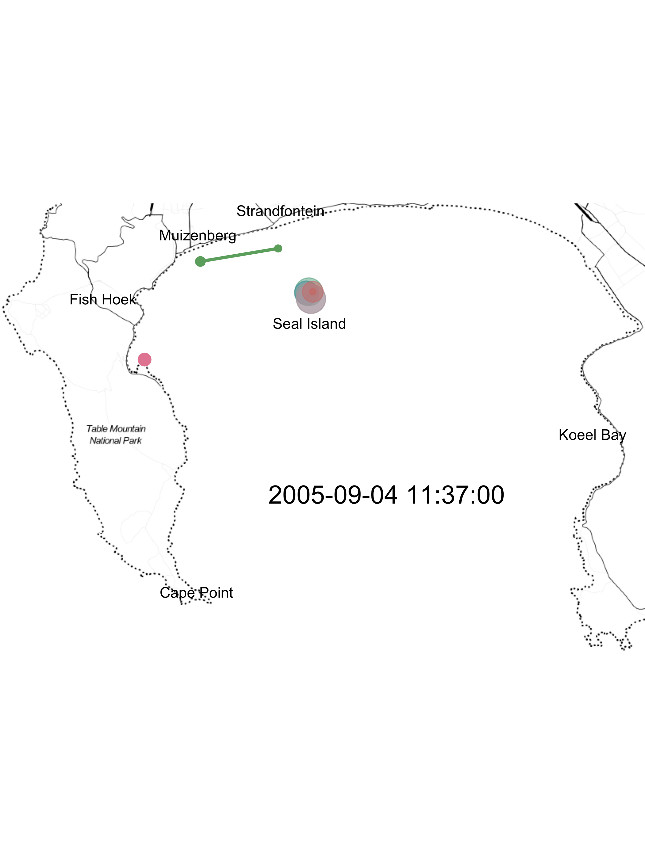

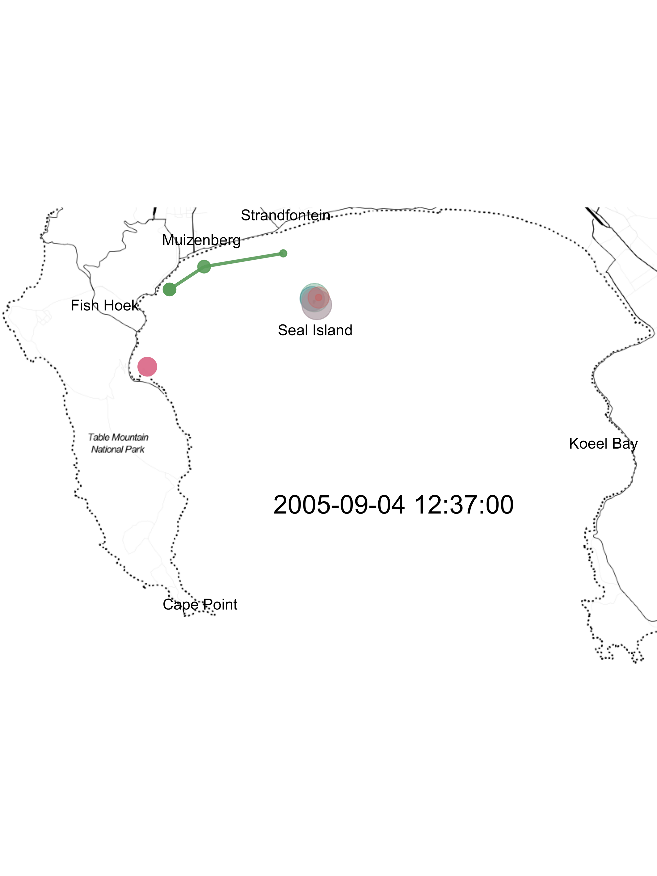


Figure 1. Two illustrative frames of the accompanying detection visualization video. Each frame summarizes detections and transitions over the previous six-hour period.
